# Single-nucleotide polymorphism of Exo1 gene is associated with risk of colorectal cancer based on robust Bayesian approach 

**Published:** 2018

**Authors:** Maryam Nasserinejad, Mohamad Amin Pourhoseingholi, Sama Rezasoltani, Setareh Akbari, Ahmad Reza Baghestani, Sadjad Shojaee, Mohammad Yaghoob-Taleghani, Ehsan Nazemalhosseini-Mojarad

**Affiliations:** 1 *Gastroenterology and Liver Diseases Research Center, Research Institute for Gastroenterology and Liver Diseases, Shahid Beheshti University of Medical Sciences, Tehran, Iran.*; 2 *Basic and Molecular Epidemiology of Gastrointestinal Disorders Research Center, Research Institute for Gastroenterology and Liver Diseases, Shahid Beheshti University of Medical Sciences, Tehran, Iran.*; 3 *Physiotherapy Research Center, Department of Biostatistics, Faculty of Paramedical Sciences, Shahid Beheshti University of Medical Sciences, Tehran, Iran*

**To Editor**

Colorectal cancer (CRC) presents as one of the most common cancers burden worldwide. It is the third most commonly diagnosed malignancy and the fourth leading cause of cancer-related death in the world (1). In Iran, CRC is the fourth leading cause of cancer death (2). Unfortunately, not only incidence of CRC has increased in both sexes during the last decade, but also the mortality rate of CRC slightly increased (3, 4). In most cases, CRC is produced through two main molecular pathways. One inhibitory pathway created by successive mutations in tumor suppressor genes and oncogenes and the mutation pathway detected by the defect in the DNA repair mismatch pair genes (5, 6). Exonuclease 1 (Exol) is the only exonocytic involved in the human MMR system. Due to the specific role of Exol in the MMR system, this gene is a predisposing factor in CRC (7-9). 

There are some different reasons that can cause the data of study to be misclassified, the reasons of misclassification can depends on type of data collection or even sample size (10) to laboratory and clinical testing Items. In this kind of data, because the sensitivity and specificity of present test are not 100%, it would be the potential of misclassification errors. Akbari* et al.* without considering of misclassification, found no significant association between EXO1 K589E (rs1047840) alleles and genotypes and risk of CRC (11). Here, it was proposed to use a new method to adjustment the misclassification, then obtain odds ratio (OR) on the same data which they used before. In this approach, robust Bayesian analysis was applied (12). In fact this method adjust the misclassification by using the sensitivity and specificity of PCR-RLFP test, and then corrects OR. As stated before, the sensitivity and specificity of RFLP-PCR is not 100% and there is no gold standard for this genetic test, so no certain values for the sensitivity and specificity of RFLP-PCR test has been reported and there is no consensus on them. On the other hand, in order to adjustment misclassified exposure and correct the OR, for using the Bayesian modeling and Sensitivity Analysis, the sensitivity, specificity and prevalence of rs1047840 SNP (Glu>Lys) in healthy subjects must be considered as the prior variables, and certain values ​​for them are included in the model. But due to the lack of certain values for the sensitivity and specificity of test, this is not possible to run. As a result, it was decided to apply a more flexible approach. 

The robust Bayesian approach acts like the Bayesian approach, with the difference that it is not necessary to consider a certain value for the prior variables and sufficient to determine the only feasible regions for the prior variables. Finally, this model also provides a feasible regions for OR, which every point of this regions can be reported, the most neutral of which is the middle of the regions (13).

According to the studies conducted for the sensitivity and specificity, it was chosen (0.75, 0.80) and (0.80, 0.90) for the sensitivity and specificity of PCR-RLFP test and for the prevalence of Glu> Lys, also it was arrived at (0.38, 0.48). θ is considered to be the log odds ratio. To perform robust Bayesian analysis (12), normal prior distribution with the mean of 0 and the variance of 0.5 was assumed for the θ. It was conducted all analyses by using R software, version 3.3.2 and Matlab software.

With considering the potential misclassification and using robust Bayesian approach, we arrived at (-0.507, 1.354) for log OR and (0.6, 3.87) for OR. The point 1.63 is the middle of the region and considered as OR.

As it is clear from the result, odds ratio are very affected by the misclassification, this means that the impact of the exposure variable was different in adjusting and without adjusting misclassification. The result of the robust Bayesian approach, according to the chosen range for prior parameters, we arrived at 1.63 for OR which means that rs1047840 SNP increased the risk CRC 1.63 times more, compared to subjects without rs1047840 SNP.

Actually, in regard to find potential predisposing risk factors of CRC, it was selected a principal polymorphism of Exo1 gene and researched whether the Exo1 K589E polymorphism could have an effect on susceptibility to CRC in an Iranian population. 

In fact Exonuclease1 (Exo1) is a member of the RAD2 nuclease family that is contributed in mismatch repair (MMR) system and play important role in cell cycle arrest mediation, establishment of genomic stability and modulation of DNA recombination. K589E (rs1047840) may replace risk of cancer as a potentially functional polymorphism in Exo1 gene by influencing the repair activity of that (14, 15). The Exo1 K589E (rs1047840) polymorphism is a non-synonymous SNP which is located on exon12 of the Exo1 gene. The present polymorphism causes dramatic amino acid alteration from a negatively charged glutamate to a positively charged lysine residue in codon 589, In this way, possibly affecting the protein functions (11). In addition, because Glu589Lys located at an exonic splicing enhancer (ESE) region, it might impact on the production of Exo1 mRNA (16).

Surprisingly, in present study we found that variant genotypes of K589E were associated with risk of CRC by applying robust Bayesian approach as the same as numerous studies which have demonstrated several SNPs of Exo1 as genetic risk factors of cancer (14, 16) for example in Taiwanese and Chinese population it was obtained that the A allele of this polymorphism has a significant association and increase the risk of lung cancer (17, 18). In contrast of present study, based on our previous findings in 2014, we did not find any correlation between rs1047840 SNP and risk of CRC by PCR-RFLP analysis (11) as well as Zienolddiny *et al.* that have presented no association between Exo1 K589E polymorphism and the risk of non-small cell lung cancer in Caucasian Norwegian (19). Also another study which was reported in Chinese population that the A allele of K589E polymorphism increases cervical cancer risk (18). 

Therefore, it can be imagined that variation of distribution frequency of Exo1 K589E genotypes may be due to carcinogenesis differences pathways among different types of tissues (11).

The present study indicated that the Exo1 K589E polymorphism have significantly critical role in genetic susceptibility to CRC based on robust Bayesian approach while there was not obtained significant association based on our previous findings in 2014 by PCR-RFLP analysis. Also it could be concluded due to the differences in the pathways of carcinogenesis among different types of tissues, differences distribution frequency of Exo1 K589E genotypes may be achieved. Also understanding the biological mechanism of the present polymorphism may lead to targeted prognosis and development of novel therapeutic strategies. 

## Conflict of interests

The authors declare that they have no conflict of interest.
